# Impact of Surgical Timing on Hospital Length of Stay Following Hemiarthroplasty and Internal Fixation for Hip Fractures: A Six-Year Retrospective Cohort Study

**DOI:** 10.3390/jcm15145484

**Published:** 2026-07-13

**Authors:** Ali H. Alyami, Abdalmalik T. Malki, Alaa Althubaiti, Hamed W. Babtain, Hasen H. Aljadani, Awwab A. Shahhatalsayed, Rafy S. Alharthi, Deemah M. Alharbi, Mohamad W. Bader

**Affiliations:** 1 College of Medicine, King Saud bin Abdulaziz University for Health Sciences, P.O. Box 9515, Jeddah 21423, Saudi Arabia; alyamia@yahoo.com (A.H.A.); thubaitia@ksau-hs.edu.sa (A.A.); babtain20063@ksau-hs.edu.sa (H.W.B.); aljadani20062@ksau-hs.edu.sa (H.H.A.); shahhat20030@ksau-hs.edu.sa (A.A.S.); alharthi20081@ksau-hs.edu.sa (R.S.A.); alharbi20072@ksau-hs.edu.sa (D.M.A.); bader20232@ksau-hs.edu.sa (M.W.B.); 2 King Abdullah International Medical Research Center, Jeddah 21423, Saudi Arabia; 3 Department of Orthopedic Surgery, Ministry of the National Guard–Health Affairs, Jeddah 21423, Saudi Arabia

**Keywords:** hip fracture, surgical timing, length of stay, hemiarthroplasty, internal fixation, elderly patients, hip fracture surgery

## Abstract

**Background:** Hip fractures are a major cause of morbidity and healthcare utilization among elderly patients. Early surgical intervention has been associated with improved postoperative outcomes; however, the effect of surgical timing on hospital length of stay (LOS) remains debated, especially in the Middle Eastern region. This study aimed to evaluate the impact of surgical timing on LOS and postoperative outcomes among elderly patients undergoing hip fracture surgery. **Methods:** A retrospective cohort study was conducted at King Abdulaziz Medical City in Jeddah, Saudi Arabia. Patients aged ≥ 60 years who underwent surgical management for proximal femur fractures between May 2019 and May 2025 were included. Surgical timing was categorized as early (<48 h from admission) or delayed (≥48 h). The primary outcome was hospital LOS. Secondary outcomes included ICU admission, venous thromboembolism (VTE), respiratory infection, periprosthetic infection, and one-year mortality. Kaplan–Meier survival analysis and Cox proportional hazards modeling were used to assess time to hospital discharge. Relative risks (RR) with 95% confidence intervals (CI) were calculated for postoperative outcomes. **Results:** A total of 179 patients were included, of whom 100 (55.9%) underwent early surgery, and 79 (44.1%) underwent delayed surgery. The median LOS was significantly shorter in the early surgery group compared with the delayed surgery group (7 days [95% CI: 6–7] vs. 11.5 days [95% CI: 9–14]; log-rank *p* < 0.001). After adjustment for comorbidities, surgical factors, and pre-hospital delay, early surgery remained independently associated with earlier discharge (hazard ratio 2.17, 95% CI 1.55–3.04; *p* < 0.001). ICU admission occurred less frequently in the early surgery group (6.0%) compared with the delayed surgery group (17.7%) (RR 0.34, 95% CI 0.14–0.83; *p* = 0.013). No significant differences were observed in VTE, respiratory infection, periprosthetic infection, or one-year mortality between groups. **Conclusions:** Surgery performed within 48 h of admission was associated with significantly shorter hospital LOS and lower ICU admission rates among elderly patients undergoing hip fracture surgery. These findings highlight the importance of timely surgical intervention to improve perioperative efficiency and hospital resource utilization.

## 1. Introduction

Hip fractures are partial or complete breaks of the femur at its junction with the pelvic bone, forming the hip joint [1]. They can be categorized according to their relationship with the hip capsule as either intracapsular fractures, involving the femoral head or femoral neck, or extracapsular fractures, including intertrochanteric, trochanteric, or subtrochanteric fractures [2].

The peak incidence of hip fractures is typically observed in individuals above the age of 70 years, with females being more susceptible than males [3]. These fractures predominantly occur in the elderly population and are usually caused by low-energy mechanisms, such as falls from standing height, which are strongly associated with osteoporosis [4]. In contrast, hip fractures in younger individuals are more commonly the result of high-energy trauma, such as motor vehicle accidents [4]. Hip fractures are associated with substantial morbidity, mortality, and healthcare costs, representing a major public health challenge worldwide [3,5]. Although geographic variation exists in the incidence of hip fractures, their clinical and economic burden remains considerable across healthcare systems [6].

Given the substantial burden of hip fractures, timely surgical intervention is considered a cornerstone of management. Previous studies have shown that early surgery is associated with shorter hospital length of stay (LOS), reduced healthcare costs, and improved postoperative recovery, whereas delayed surgery has been associated with increased postoperative complications and mortality [7,8,9,10,11,12,13,14]. However, in Saudi Arabia, evidence remains limited, with only one published study evaluating the effect of surgical timing on hip fracture outcomes, focusing primarily on mortality [15]. Consequently, locally relevant evidence regarding the association between surgical timing and hospital LOS remains scarce.

Hospital LOS is an important indicator of postoperative recovery and healthcare efficiency, as prolonged hospitalization increases healthcare costs, resource utilization, and demand on hospital services [16]. Despite growing international evidence evaluating the relationship between surgical timing and postoperative outcomes, relatively few studies have focused on LOS as the primary outcome.

Nevertheless, the reported benefits of early surgery remain inconsistent across studies, and the optimal timing of surgical intervention continues to be debated [12,15,17]. Therefore, this study aimed to evaluate the impact of surgical timing on hospital LOS and selected postoperative outcomes among elderly patients undergoing hip fracture surgery by comparing patients treated within 48 h of admission with those treated after 48 h.

## 2. Methods

### 2.1. Study Design and Setting

This retrospective cohort study was conducted at King Abdulaziz Medical City, a level I trauma center in Jeddah, Saudi Arabia, using data from the BEST Care Health Information System, the electronic medical record platform of the Ministry of National Guard Health Affairs. BEST Care provides standardized and comprehensive patient information across all departments, enabling centralized data collection. In this institution, proximal femoral fractures are managed as urgent or emergency conditions, and institutional policy aims to perform surgery within 24 h of admission whenever clinically appropriate. Delays may occur because of patient medical optimization, interfacility transfer, or competing emergency surgical demands.

### 2.2. Study Population

We included both genders aged 60 years or older who underwent surgical treatment for proximal femur fractures (femoral neck, intertrochanteric, or subtrochanteric) between May 2019 and May 2025. The age threshold of 60 years was selected to reflect the demographic characteristics and clinical practice patterns of our region. In this trauma center, as well as in many centers across the Middle East, patients aged 60 years and older with proximal femoral fractures are commonly managed according to geriatric hip fracture treatment pathways. However, age alone did not determine the choice of surgical procedure, which was also guided by fracture pattern, pre-injury functional status, bone quality, comorbidities, and surgeon judgment. Patients were excluded if they had incomplete records, bone malignancy or pathological fractures, non-surgical management, total hip arthroplasty (THA), or revision surgery. THA is typically reserved for younger, more active patients or those with pre-existing hip arthritis, while hemiarthroplasty is preferred in frail elderly patients; including THA would therefore introduce selection bias. Similarly, revision arthroplasty cases generally involve poorer bone quality and a higher comorbidity burden, which could artificially inflate complication and mortality rates unrelated to surgical timing.

### 2.3. Data Collection

Data were extracted from BEST Care using a structured sheet covering demographic information, comorbidities, fracture characteristics, surgical details, perioperative care, and outcomes. Demographic variables included patient age at admission and sex. Baseline comorbidities were identified through documented diagnoses in the medical record and included hypertension, diabetes mellitus, ischemic heart disease, stroke, chronic kidney disease, hypothyroidism, heart failure, Parkinson’s disease, neurodegenerative disease, obesity, dyslipidemia, osteoporosis, and history of previous fractures. These comorbidities were recorded to account for underlying health conditions that may influence surgical timing and postoperative outcomes.

Fracture characteristics were determined from radiology reports and operative documentation. Fractures were anatomically classified as femoral neck, intertrochanteric, and subtrochanteric fractures. Additional perioperative information was collected from surgical and anesthetic records. Surgical details included the type of surgical procedure performed (internal fixation or hemiarthroplasty), type of anesthesia administered (general or spinal anesthesia), and the use of preoperative imaging studies used for fracture evaluation and surgical planning. The timing of surgery was categorized as early (<48 h from admission) or delayed (≥48 h).

All eligible patients undergoing surgical management for proximal femur fractures who met the predefined inclusion criteria between May 2019 and May 2025 were consecutively enrolled. This approach minimized selection bias by ensuring that every eligible patient during the study period was included.

### 2.4. Outcomes

The primary outcome of interest was hospital LOS, defined as the total number of days from hospital admission to hospital discharge. LOS was calculated using admission and discharge timestamps recorded in the electronic health record system. Postoperative LOS was defined as the interval from surgery to hospital discharge or in-hospital death. Secondary outcomes included postoperative complications and clinical events occurring after surgery, including respiratory infections, periprosthetic or fracture-related infections, venous thromboembolism (VTE), intensive care unit (ICU) admission, and mortality.

Periprosthetic or fracture-related infections were identified according to the internationally accepted consensus definition of fracture-related infection (FRI), including both confirmatory and suggestive criteria as documented in the medical records. Respiratory infections were identified based on documented physician diagnoses supported by clinical assessment, radiological findings, and microbiological testing when available. Venous thromboembolism events included both deep vein thrombosis (DVT) and pulmonary embolism (PE) and were recorded when confirmed by imaging studies, including Doppler ultrasonography for DVT or computed tomography pulmonary angiography for PE, as documented in the medical record. ICU admission was defined as any postoperative transfer to the intensive care unit during the index hospitalization, regardless of the patient’s immediate postoperative destination following surgery, and it was verified through hospital admission records and patient flow documentation within the electronic health record. Mortality status was determined using documented hospital records and follow-up information available within the electronic health system. All outcomes were assessed up to one year after surgery. For survival analysis, follow-up time was calculated from the day after surgery until death or completion of the one-year follow-up period. Given the retrospective design of the study, formal adjudication of ambiguous cases was not performed, and outcome ascertainment relied on contemporaneous physician documentation within the electronic medical record.

### 2.5. Confidentiality and Data Protection

Patient confidentiality was strictly maintained throughout the study. No patient identifiers were collected at any stage of data collection. All data were anonymized, securely stored in password-protected systems, and handled in accordance with institutional data protection policies. Access to the data was restricted exclusively to the research team.

### 2.6. Ethical Approval

The study protocol was reviewed and approved by the Institutional Review Board (IRB) of the Ministry of National Guard Health Affairs (NRJ25/053/4). The study was conducted in full compliance with internationally accepted ethical standards for medical research involving human participants and was approved.

### 2.7. Statistical Analysis

Data were presented as medians and interquartile ranges (IQRs) for continuous variables, as they were non-normally distributed. Frequencies and percentages were used to describe categorical variables. The Chi-square test or Fisher’s exact test was used to compare categorical data. The Wilcoxon rank-sum test was applied to compare two independent groups with continuous variables. Results were expressed as relative risk (RR) with 95% confidence intervals (CI). A *p*-value ≤ 0.05 was considered statistically significant. Kaplan–Meier survival analysis with the log-rank test was used to compare time to hospital discharge between early and delayed surgery groups. Multivariable Cox proportional hazards regression was performed to identify factors independently associated with time to hospital discharge. Statistical analyses were performed using JMP Pro 18 software (SAS Institute Inc., Cary, NC, USA).

## 3. Results

Among the 179 patients included in this cohort, 100 (55.9%) underwent early surgery, while 79 (44.1%) underwent delayed surgery. Most patients were female (56.4%). The median age was 75 years (IQR: 69–82). Hypertension was the most prevalent comorbidity, affecting 75.9% of the patients, while Parkinson’s disease was the least commonly reported, occurring in 4.5% of the patients. Pre-hospital delay was comparable between the study groups, with no significant difference observed between patients undergoing early and delayed surgery (*p* = 0.953) (Table 1).

Regarding fracture characteristics, intertrochanteric fractures were the most frequent fracture type in the early surgery group (53%), whereas femoral neck fractures were more commonly observed in the delayed surgery group (59.5%). Subtrochanteric fractures were relatively uncommon in both groups (5% in early vs. 3.8% in delayed). Although distribution patterns differed between groups, this difference was not statistically significant (*p* = 0.37). Closed reduction and internal fixation were the most performed surgical procedures across both groups (51.0% in early vs. 34.2% in delayed), followed by hemiarthroplasty (40.0% vs. 46.8%), with a statistically significant difference in surgical procedure distribution between early and delayed surgery groups (*p* = 0.036) (Table 2). Reasons for delayed surgery are presented in Table 3, with medical optimization representing the most common cause of operative delay.

**Figure 1:**
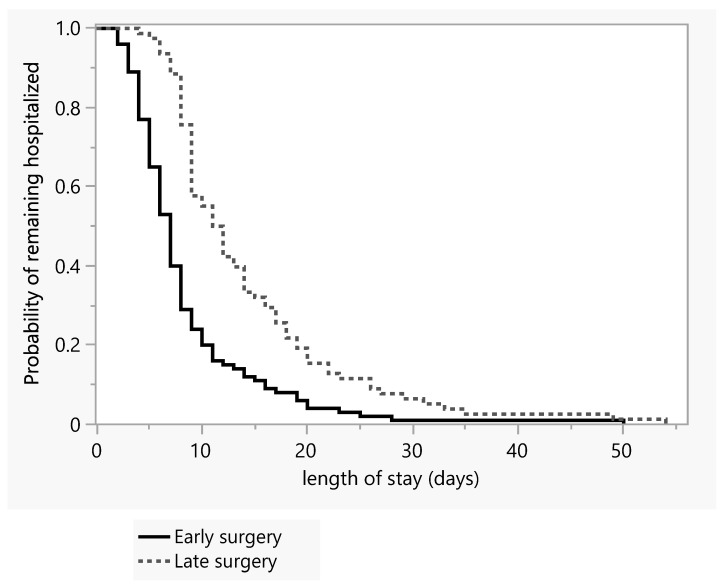
Kaplan–Meier curve for time to hospital discharge according to surgical timing.

**Figure 2:**
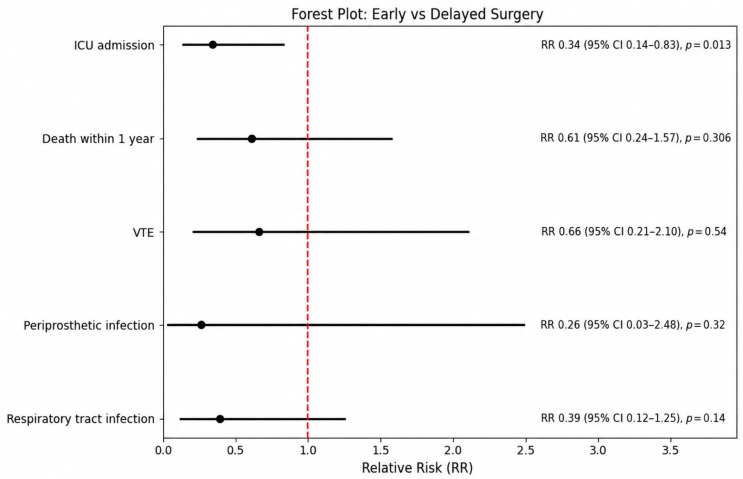
Forest plot illustrating the relative risk (RR) of postoperative outcomes comparing early (<48 h) versus delayed (≥48 h) hip fracture surgery. The dashed vertical line represents the line of no effect (RR = 1). Points represent the estimated relative risks, and horizontal lines represent the 95% confidence intervals.

Kaplan–Meier analysis demonstrated that the median length of hospital stay was significantly shorter in the early surgery group compared with the late surgery group (7 days [95% CI: 6–7] vs. 11.5 days [95% CI: 9–14]; log-rank *p* < 0.001) (Figure 1). A sensitivity analysis excluding two patients who died before hospital discharge yielded similar findings, with the median hospital LOS remaining 7 days (95% CI: 6–7) in the early surgery group and 12 days (95% CI: 9–14) in the delayed surgery group (log-rank *p* < 0.001). Results of the multivariable Cox proportional hazards model are presented in Table 4. After adjustment for hypertension, ischemic heart disease, previous stroke, chronic kidney disease, surgery type, osteoporosis, and pre-hospital delay, early surgery remained independently associated with earlier hospital discharge (adjusted HR 2.17, 95% Cl 1.55–3.04; *p* < 0.001). None of the remaining covariates, including pre-hospital delay, were independently associated with time to hospital discharge. A secondary analysis of postoperative length of stay also demonstrated a significantly shorter postoperative hospitalization among patients undergoing early surgery (median 5 days [IQR 3.25–8] vs. 6 days [IQR 4–10]; Wilcoxon *p* = 0.025). Furthermore, ICU admission occurred more frequently in the delayed surgery group (17.7%) compared with the early surgery group (6.0%). This difference was statistically significant, χ^2^ (1, N = 179) = 6.11, *p* = 0.013. Patients undergoing early surgery had a significantly lower risk of ICU admission compared with those undergoing delayed surgery (RR = 0.34, 95% CI [0.14, 0.83]) (Figure 2).

As for mortality, within one year, 7 patients (7.0%) in the early surgery group and 9 patients (11.4%) in the delayed surgery group died. Early surgery was associated with a lower risk of one-year mortality compared with delayed surgery (RR = 0.61, 95% CI [0.24, 1.57]); however, this difference did not reach statistical significance [χ^2^ (1, N = 179) = 1.046, *p* = 0.306] (Figure 2).

Moreover, the incidence of VTE was low in both groups, occurring in 5 patients (5.0%) in the early surgery group and 6 patients (7.6%) in the delayed surgery group. There was no statistically significant association between surgical timing and the occurrence of VTE (*p* = 0.54). Although the risk of VTE was lower in the early surgery group compared with the delayed group (RR = 0.66, 95% CI [0.21, 2.10]), this difference did not reach statistical significance (Figure 2). Among the 11 patients who developed VTE, most had at least one established thromboembolic risk factor. The most frequently identified risk factors were immobility related to fracture, trauma, or surgery (6/11, 54.5%), followed by metabolic or obesity-related factors (5/11, 45.5%) and cardiovascular or renal comorbidities (3/11, 27.3%). Malignancy and a history of previous thrombosis were each present in 2 patients (18.2%), while hormonal risk factors were identified in 1 patient (9.1%).

Periprosthetic infection occurred in 1 of 100 patients (1.0%) in the early surgery group and in 3 of 79 patients (3.8%) in the delayed surgery group. There was no statistically significant association between surgical timing and periprosthetic infection (Fisher’s exact *p* = 0.32). Patients undergoing early surgery had a lower risk of infection compared with those undergoing delayed surgery (RR = 0.26, 95% CI [0.03, 2.48]); however, this difference did not reach statistical significance (Figure 2).

Respiratory tract infection occurred in 4 of 100 patients (4.0%) in the early surgery group and in 8 of 79 patients (10.1%) in the delayed surgery group. No statistically significant association was observed between surgical timing and respiratory tract infection (Fisher’s exact *p* = 0.14). The relative risk of respiratory tract infection for early versus delayed surgery was 0.39 (95% CI [0.12, 1.25]), suggesting a lower risk in the early group, although this difference was not statistically significant (Figure 2).

## 4. Discussion

This study evaluated the impact of surgical timing on hospital length of stay as the primary outcome, as well as postoperative morbidity and mortality, among elderly patients undergoing hemiarthroplasty or internal fixation for hip fractures at a tertiary care center in Saudi Arabia. The results showed that, compared with earlier intervention, surgery performed after 48 h was linked to a noticeably longer LOS. Furthermore, delays in surgery were also linked to an increased risk of ICU admission. However, there were no appreciable variations between the groups in terms of postoperative infections, VTE, or one-year mortality. These results highlight the critical role of surgical timing in determining hospitalization duration and perioperative resource utilization, even if it may not have a direct impact on long-term survival.

Our findings regarding length of hospital stay are consistent with those reported by Lefaivre et al. [11], who demonstrated that surgical delay independently prolonged time to discharge using Cox proportional hazards modeling. In our cohort, surgery beyond 48 h was likewise associated with significantly longer hospitalization, with early intervention reducing median LOS from 11.5 to 7 days and remaining an independent predictor of earlier discharge after adjustment for comorbidities. Notably, Lefaivre et al. also found no independent association between surgical delay and mortality, paralleling our results. Similar patterns have been observed in other studies. Unnanuntana et al. [8] and Dong et al. [14] reported shorter median LOS among patients treated within 24 h compared with those undergoing surgery after 24 h, despite using a different timing threshold. Likewise, Siegmeth et al. [18], in a large prospective cohort of 3,628 patients, demonstrated that surgery beyond 48 h significantly prolonged hospitalization (21.6 vs. 36.5 days), with each 7.85-h delay corresponding to one additional inpatient day. Although absolute hospital LOS differs across healthcare systems, the observed association between earlier surgery and shorter hospitalization appears consistent across studies. Nevertheless, differences in healthcare organization, referral pathways, and resource availability between Saudi Arabia and previously studied healthcare systems should be considered when comparing absolute LOS estimates and interpreting the generalizability of these findings. Furthermore, comparable findings were also reported by Orosz et al. [19], who showed that early surgery reduced LOS by approximately 1.9 days without conferring a survival advantage. In our cohort, the reduction in LOS was even more pronounced, with an adjusted hazard ratio of 2.17 for earlier discharge. Importantly, pre-hospital delay did not differ significantly between the early and delayed surgery groups and was not independently associated with time to hospital discharge in the multivariable analysis, suggesting that differences in hospital LOS were unlikely to be explained by delayed presentation following injury. While Orosz et al. [19] additionally reported reduced pain and fewer major complications in restricted analyses, we observed lower ICU admission rates among patients treated within 48 h, despite no significant differences in one-year mortality, VTE, or infection. The absence of a significant association between surgical timing and VTE should be interpreted cautiously, as most patients who developed VTE had one or more established thromboembolic risk factors, suggesting that patient-related clinical characteristics may have contributed more substantially to VTE occurrence than surgical timing alone. The association between surgical delay and prolonged hospitalization may be explained by both physiological and system-level mechanisms [20]. Prolonged immobilization prior to surgery can contribute to deconditioning, increased risk of postoperative complications, pulmonary compromise, and delayed postoperative mobilization, all of which may extend recovery time [19,21]. Our additional analysis demonstrated that most delayed procedures were attributable to medical optimization or anticoagulant management, whereas only a small proportion resulted from logistical factors. These findings suggest that many surgical delays reflected clinical optimization needs rather than predominantly avoidable system-related delays and should therefore be considered when interpreting the observed association between surgical timing and hospital LOS [22].

Most of the conflicting findings in the literature relate to mortality. In contrast to our results, several studies have reported an association between surgical delay and increased mortality. Tran et al. [7], in a national analysis, demonstrated that delays beyond 48 h significantly increased both mortality and complication rates. Similarly, Dong et al. [14] reported higher mortality among patients undergoing surgery after 24 h, although they also observed prolonged hospitalization in delayed cases, consistent with our LOS findings. Furthermore, Moja et al. [10] identified a significant association between delayed surgery and increased mortality risk, underscoring the time-sensitive nature of hip fracture management. This variability in mortality findings across studies may reflect differences in study design, adjustment strategies, patient populations, and definitions of early versus delayed surgery. Large registry-based and pooled analyses often include patients with greater baseline medical instability and comorbidity burden, both independent predictors of mortality, raising the possibility of residual confounding despite statistical adjustments [23]. Moreover, the use of longer-term mortality endpoints, as in our study, may attenuate short-term survival differences attributable to surgical timing alone. Collectively, these findings suggest that, while mortality outcomes remain debated, the most consistent and reproducible impact of surgical timing in hip fracture patients is observed in discharge efficiency and perioperative morbidity.

The findings of this study should be interpreted in the context of potential confounding by indication. Patients undergoing delayed surgery often require additional medical optimization because of more severe cardiovascular, renal, or other systemic comorbidities that may independently influence postoperative outcomes. Although our multivariable analysis adjusted for major comorbidities, residual confounding from differences in disease severity and the specific clinical circumstances leading to surgical delay cannot be excluded. Consequently, the observed associations may partially reflect appropriate clinical decision-making rather than the independent effect of surgical timing alone.

Furthermore, the retrospective observational design introduces the possibility of immortal time bias, as patients in the delayed-surgery group were required to survive long enough to undergo surgery. Although this limitation is inherent to retrospective studies evaluating surgical timing, it should be considered when interpreting the relationship between operative delay and clinical outcomes.

### 4.1. Strengths

This study has several important strengths. To our knowledge, it is the first study in Saudi Arabia to specifically evaluate the association between surgical timing and hospital LOS as a primary outcome among elderly patients undergoing surgery for hip fractures and only the second nationally to examine the broader impact of surgical timing on clinical outcomes in this population. By focusing on LOS as a systems-level endpoint, the study addresses a clinically and operationally meaningful outcome that has direct implications for healthcare resource utilization and patient flow within tertiary care settings. Methodologically, the use of Kaplan–Meier survival analysis and multivariable Cox proportional hazards modeling provides a robust and appropriate framework for evaluating time-to-discharge, allowing adjustment for key comorbidities and reducing confounding effects. The inclusion of a consecutive five-year cohort from a large tertiary care center enhances the representativeness of the sample and reflects real-world clinical practice. Additionally, the use of a standardized electronic medical record system ensured comprehensive data capture and consistent documentation of surgical timing and postoperative outcomes. Collectively, these features strengthen the internal validity and practical relevance of the findings.

### 4.2. Limitations

This study has several limitations. First, its retrospective single-center design limits causal inference. Second, the relatively small sample size and the low frequency of several secondary outcomes, including VTE and postoperative infections, limited the statistical power to detect significant differences. Accordingly, analyses of these secondary outcomes should be considered exploratory and interpreted with caution. Additionally, because patients in the delayed-surgery group had longer hospital stays, they may have had a greater opportunity for postoperative complications to be detected and documented, introducing potential detection bias. Finally, the study period overlapped with the COVID-19 pandemic, and although emergency hip fracture services were maintained throughout this period, the indirect effects of the pandemic on healthcare delivery and patient outcomes cannot be completely excluded.

## 5. Conclusions

In conclusion, surgical timing significantly influences hospital length of stay among elderly patients undergoing hemiarthroplasty or internal fixation for hip fractures. Surgery performed within 48 h of admission was independently associated with earlier discharge and a lower risk of ICU admission, highlighting its importance in improving perioperative efficiency and resource utilization. However, no significant differences were observed in one-year mortality, VTE, or infection rates. These findings suggest that the principal benefit of timely hip fracture surgery lies in optimizing inpatient recovery and healthcare system performance rather than conferring a clear survival advantage. Further multicenter prospective studies are warranted to validate these findings and refine timing thresholds within the local context.

## Figures and Tables

**Table 1:** Baseline Demographic and Clinical Characteristics of Early and Late Surgery Groups.

Variable	Early Surgery (*n* = 100)	Late Surgery (*n* = 79)	*p*-Value
Gender			0.633
Male	42 (42%)	36 (45.6%)	
Female	58 (58%)	43 (54.4%)	
Age, years,			0.523
Median (IQR)	75 (69.3–81.8)	75 (67–82)	
Pre-hospital LOS			0.953
<1 day	45 (45%)	37 (46.8%)	
1–2 days	33 (33%)	26 (32.9%)	
≥3 days	22 (22%)	16 (20.3%)	
Body mass index, kg/m^2^, Median (IQR)	25.4 (21.8–28.8)	25.6 (22.9–30.8)	0.276
Previous hip surgery	8 (8%)	7 (8.9%)	0.837
Hypertension	69 (69%)	67 (84.8%)	0.014
Diabetes mellitus	55 (55%)	52 (65.8%)	0.143
Ischemic heart disease	18 (18%)	24 (30.4%)	0.052
Previous cerebrovascular accident (stroke/TIA)	17 (17%)	25 (31.6%)	0.022
Chronic kidney disease	14 (14%)	21 (26.6%)	0.035
Hypothyroidism	7 (7%)	6 (7.6%)	0.879
Heart failure	5 (5%)	5 (6.3%)	0.701
Parkinson disease	5 (5%)	3 (3.8%)	0.699
Neurodegenerative disease	11 (11%)	7 (8.9%)	0.637
Dyslipidemia	23 (23%)	16 (20.3%)	0.658
Osteoporosis (DEXA-confirmed)	17 (17%)	4 (5.1%)	0.014
Previous osteoporotic fracture	6 (6%)	9 (11.4%)	0.20

Data are presented as *n* (%) for categorical variables and median (interquartile range [IQR]) for continuous variables. Comparisons between groups were performed using the Chi-square test or Fisher’s exact test for categorical variables and the Mann–Whitney U test for continuous variables. A *p*-value < 0.05 was considered statistically significant. DEXA = dual-energy X-ray absorptiometry; TIA = transient ischemic attack.

**Table 2:** Fracture and Surgical Characteristics According to Surgical Timing.

Fracture and Surgical Details	Early Surgery (*n* = 100)	Late Surgery (*n* = 79)	*p*-Value
Mechanism of injury: Mechanical fall Other	93 (93%) 7 (7%)	72 (91.1%) 7 (8.9%)	0.645
Advanced imaging obtained for fracture evaluation (CT/MRI)	19 (19%)	21 (26.6%)	0.227
Fracture site * NOF Intertrochanteric Subtrochanteric	49 (49%) 53 (53%) 5 (5%)	47 (59.5%) 34 (43%) 3 (3.8%)	0.370
Fracture side Left Right Bilateral	49 (49%) 50 (50%) 1 (1%)	36 (45.6%) 43 (54.4%) 0 (0%)	0.490
Surgery type CRIF Hemiarthroplasty ORIF	51 (51%) 40 (40%) 9 (9%)	27 (34.2%) 37 (46.8%) 15 (19%)	0.036
Type of anesthesia General Spinal	93 (93%) 7 (7%)	72 (91.1%) 7 (8.9%)	0.544
Immediate postoperative destination ** Ward ICU	96 (96%) 4 (4%)	74 (93.7%) 5 (6.3%)	0.210

NOF = neck of femur; CRIF = closed reduction internal fixation; ORIF = open reduction internal fixation; CT = computed tomography; MRI = magnetic resonance imaging; ICU = intensive care unit. * Fracture site categories are not mutually exclusive because some patients had fractures involving more than one anatomical region; therefore, totals exceed the number of patients. ** Immediate postoperative destination refers to the patient’s initial location immediately following surgery and does not represent ICU admission at any time during the index hospitalization.

**Table 3:** Primary reasons for delayed surgery among patients undergoing hip fracture surgery ≥48 h after hospital admission (*n* = 79).

Reason for Delayed Surgery	*n* (%)
Medical optimization	50 (63.3%)
Anticoagulant therapy	20 (25.3%)
Logistical factors *	9 (11.4%)

* Logistical factors included operating room availability and other hospital scheduling constraints.

**Table 4:** Multivariable Cox proportional hazards model for time to hospital discharge.

Variable	Adjusted HR *	95% CI	*p*-Value
Early Surgery	2.17	1.55–3.04	<0.001
Hypertension	0.85	0.58–1.25	0.407
Ischemic heart disease	0.89	0.62–1.30	0.558
Previous cerebrovascular accident (stroke/TIA)	0.95	0.65–1.38	0.778
Chronic kidney disease	0.81	0.53–1.24	0.329
Osteoporosis (DEXA-confirmed)	1.24	0.88–1.74	0.223
Surgery type			0.338
CRIF	Reference **	-	
ORIF	1.16	0.71–1.90	
Hemiarthroplasty	0.86	0.61–1.20	
Pre-hospital LOS			0.311
<1 day	Reference ***	-	
1–2 days	0.95	0.66–1.35	
≥3 days	0.68	0.45–1.03	

* Hazard ratios (HRs) greater than 1 indicate a higher probability of earlier hospital discharge. ** Reference category: CRIF. Hazard ratios for ORIF and hemiarthroplasty are estimated relative to CRIF. *** Reference category: Pre-hospital LOS < 1 day. Hazard ratios for the remaining categories are estimated relative to <1 day.

## Data Availability

Datasets are available from the corresponding author on reasonable request.

## Abbreviations

The following abbreviations are used in this manuscript:

LOS	Length of stay
ICU	Intensive care unit
VTE	Venous thromboembolism
DVT	Deep venous thrombosis
PE	Pulmonary embolism
